# Long‐Term Human Skin Platform for Modeling Chronic Inflammation, Environmental Stress, and Therapeutic Intervention

**DOI:** 10.1002/advs.78035

**Published:** 2026-09-29

**Authors:** Purnendu Kumar Sharma, Evelien Schaafsma, Elysia Anderson, Jocelyn Chiari, Paige Thompson, Erin Galt, Sunghoon Lee, Jason Holsapple, Sai Hein, Bailey L. Coates, Jade Michaud, Sabrina Zuccaro, Lia Kent, Chris Hinojosa, Kyung‐Jin Jang

**Affiliations:** ^1^ Outer Biosciences, Inc. Malden Massachusetts USA

**Keywords:** cellular senescence, chronic inflammation, ex vivo human skin model, inflammaging, long‐term culture, senolytics, UV‐induced damage

## Abstract

Chronic inflammation drives tissue dysfunction and aging, yet the dynamic interplay between persistent inflammatory signaling and structural deterioration remains difficult to study in human‐relevant systems. Here, an advanced long‐term human skin platform is presented that preserves native tissue architecture and epidermal, stromal, and immune‐associated molecular programs for up to 4 weeks. Time‐course RNA sequencing of 199 tissue samples from 20 donors revealed an early culture adaptation phase followed by stabilization of tissue‐associated transcriptional programs. Using this system, sustained IL‐17/IL‐22 stimulation over 3 weeks induced chronic inflammatory transcriptional programs, progressive histopathological changes, and persistent secretion of IL‐8, MCP‐1, S100A8/A9, and MMP‐1, which was broadly suppressed by the Janus kinase inhibitor tofacitinib. In aged donor tissue, prolonged senolytic‐associated treatment attenuated inflammatory and remodeling pathways and reduced senescence‐associated secretory factors, including IL‐6 and MMP‐1, with significant treatment‐associated effects evident at Week 3 but not Week 1. Finally, ultraviolet B exposure triggered coordinated stress and inflammatory responses that were mitigated using topical sunscreen, demonstrating compatibility with environmental stress modeling and topical intervention. Together, these findings establish a versatile human skin platform for modeling chronic inflammation, aging‐associated tissue remodeling, and environmental stress, providing a translational framework for investigating skin tissue dysfunction and evaluating therapeutic interventions.

## Graphical abstract

An advanced, long‐term human skin platform maintains native skin structure and key tissue functions for up to four weeks. The platform models chronic inflammation, aging‐related skin changes, and UVB damage, enabling drug and topical interventions—including anti‐inflammatory therapy, senolytic treatment, and sunscreen—to be evaluated in intact human skin, supporting translational skin research.

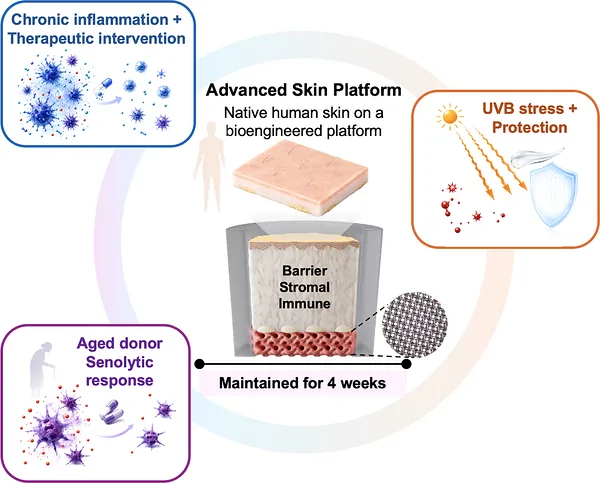

## 1 Introduction

Chronic inflammatory signaling contributes to tissue dysfunction in both disease‐associated and aging‐associated contexts. In chronic inflammatory skin diseases, persistent cytokine signaling can drive barrier disruption, immune activation, and stromal remodeling, while incomplete resolution may leave residual inflammatory gene expression and tissue‐resident immune programs in previously affected sites [1, 2, 3, 4]. In aging, chronic low‐grade inflammation, termed inflammaging, arises from cumulative cellular stress and is sustained by mechanisms including cellular senescence, the senescence‐associated secretory phenotype (SASP), and age‐associated immune dysregulation [5, 6, 7, 8, 9, 10, 11]. Although these processes are well characterized at the molecular level, how persistent inflammatory signaling translates into structural tissue deterioration over time remains incompletely understood.

Human skin represents a uniquely accessible and physiologically relevant system to study these processes. As a barrier organ, it integrates intrinsic cellular programs with continuous environmental exposures, functionally linking immune signaling, extracellular matrix (ECM) remodeling, and barrier integrity [3, 4]. During aging, epidermal, immune, and stromal compartments undergo functional reprogramming toward pro‐inflammatory and remodeling‐associated states, with keratinocyte activation and senescence‐associated secretory programs involving epidermal keratinocytes and dermal fibroblasts contributing to barrier dysfunction, matrix degradation, and tissue remodeling [8, 9, 10, 11, 12, 13, 14, 15]. These processes are further exacerbated by extrinsic stressors such as ultraviolet (UV) radiation, reinforcing the skin as a central model for studying the convergence of chronic inflammation, senescence, and environmental damage [16, 17].

Despite their importance, these complex, multi‐layered dynamics remain difficult to study using existing experimental systems. Existing human skin models include two‐dimensional keratinocyte or fibroblast cultures, reconstructed epidermal and full‐thickness skin equivalents, organotypic models, microphysiological systems, and ex vivo human skin explants. Cell‐based and reconstructed models provide experimental control and, in some cases, multi‐week culture, but often rely on selected cell types and simplified architecture that do not fully capture native donor‐specific tissue organization, resident immune/stromal complexity, or physiological barrier properties [18, 19]. While human skin explants preserve native tissue complexity, conventional explant culture is often limited by progressive culture‐associated changes and declining tissue integrity over time [20]. Although extended ex vivo culture protocols have reported maintenance of human skin explants for several weeks [21], comprehensive longitudinal validation across multiple donors integrating histological, transcriptomic, and functional readouts remains limited. Animal models provide valuable insights, but they fail to fully recapitulate human‐specific immune‐stromal interactions and epidermal architecture, limiting their translational relevance [3]. These limitations highlight the need for human‐relevant systems that preserve tissue architecture and immune responsiveness over extended durations, while enabling evaluation of therapeutic strategies targeting complex and chronic tissue conditions [22, 23, 24].

Here, we present a long‐term human skin platform that preserves native tissue architecture, multicellular interactions, and molecular fidelity for up to 4 weeks in ex vivo culture. Using this system, we model key dimensions of chronic tissue stress, including sustained cytokine‐driven inflammation and its pharmacologic modulation, senolytic‐associated treatment responses in aged donor tissue, and environmental stress evaluated via topically applied interventions. This platform provides a robust and translationally relevant framework for evaluating therapeutic strategies targeting chronic skin tissue dysfunction.

## 2 Results

### 2.1 An Advanced Long‐Term Human Skin Platform Supports Extended Tissue Maintenance

To establish a system suitable for long‐term studies of complex human skin biology, we developed a bioengineered ex vivo culture platform capable of maintaining native full‐thickness human skin tissue over extended durations. The system incorporates a custom 3D‐printed insert configured for 24‐well plate use, featuring a highly porous gyroid architecture with interconnected pores (∼700 µm pores, ∼70% porosity) that supports skin tissue at an air‐liquid interface (ALI) while enabling enhanced nutrient and waste exchange from the basal compartment (Figure 1A,B).

**FIGURE 1:**
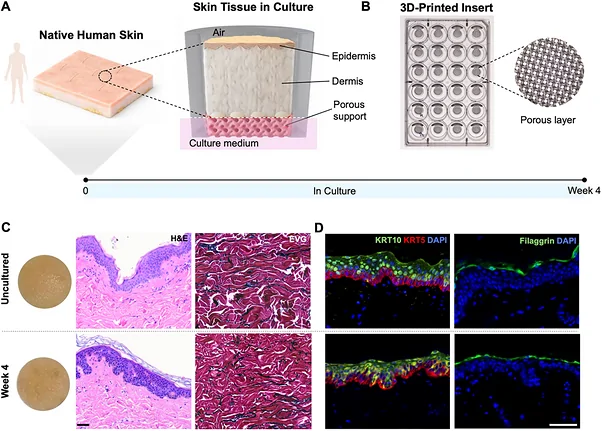
Advanced human skin platform for long‐term maintenance of human skin architecture. (A) Schematic of the long‐term human skin platform. Full‐thickness skin tissues obtained from human donors were processed to generate up to 100 individual biopsies per tissue specimen and maintained at an air‐liquid interface on a porous support platform for up to 4 weeks. (B) Top‐down photograph of 3D‐printed inserts designed to accommodate 8‐mm skin tissues in a 24‐well plate format. The gyroid porous structure consists of interconnected open pores with an average diameter of ∼700 µm and a porosity of ∼70%. (C) Representative images and histological analysis at uncultured (Day 0) and Week 4 (Day 30). Skin surface photography and H&E and EVG staining were used to assess epidermal and dermal architecture. (D) Representative IF images of epidermal markers over 4 weeks. KRT5 and KRT10 were used to assess basal and suprabasal epidermal organization, and filaggrin was used to assess terminal differentiation and barrier formation. Scale bar, 50 µm.

Human skin tissues were obtained from surgical excision, bulk‐processed to generate up to 100 individual biopsies per tissue, and cultured in the platform for up to 4 weeks. No overt macroscopic changes, including discoloration, were observed over time in untreated conditions. Histological assessment demonstrated overall preservation of tissue integrity throughout the culture period (Figure 1C). Hematoxylin and eosin (H&E) and Verhoeff's Van Gieson (EVG; collagen in red, elastin in black) staining showed that key architectural features were largely retained, including a stratified epidermis, an intact dermal–epidermal interface, and an organized dermal compartment. To further assess epidermal organization, immunofluorescence (IF) staining was performed for markers of basal keratinocytes (keratin 5, KRT5), suprabasal differentiation (keratin 10, KRT10), and terminal differentiation/barrier formation (filaggrin) (Figure 1D). These markers exhibited consistent spatial localization patterns between uncultured (Day 0) and Week 4 samples, indicating preservation of epidermal compartmentalization and differentiation states over time. To benchmark tissue maintenance against a conventional culture format, skin biopsies from the same donor were cultured in parallel using either the 3D‐printed insert platform or a conventional transwell‐based configuration. At Day 7, both culture configurations showed overall preservation of epidermal and dermal architecture. By Day 15, transwell‐cultured tissues showed marked epidermal disruption and separation, whereas tissues maintained in the 3D‐printed insert platform retained epidermal and dermal architecture (Figure S1). Together, these findings demonstrate that the platform supports multi‐week maintenance of human skin tissue while preserving overall tissue architecture, including epidermal organization and dermal structural integrity, providing a stable foundation for downstream functional and molecular analyses.

### 2.2 Longitudinal Transcriptomic Analysis Reveals Stabilization of Tissue Homeostasis Programs

We next characterized the molecular behavior of human skin maintained in the platform over four weeks using bulk RNA sequencing (RNA‐seq) across serial time points from 20 donors (Figure 2). Principal component analysis (PCA) showed clear separation between uncultured tissue and cultured samples, consistent with a pronounced early adaptation phase following tissue excision and transport. Notably, samples from Weeks 1–4 occupied a relatively compact transcriptional space rather than diverging progressively over time, indicating an early transcriptional shift followed by stabilization (Figure 2A). To obtain an overview of temporal pathway dynamics, we performed gene set enrichment analysis (GSEA) using Gene Ontology Biological Process (GOBP) terms comparing each culture week to the uncultured condition and highlighted pathways exhibiting distinct temporal patterns, including transient early responses, progressive increases over time, and sustained enrichment across the culture period (Figure 2B). These patterns illustrate dynamic transcriptional adaptations following tissue excision and culture. To more systematically characterize these dynamics, we next identified modules of co‐expressed genes with shared temporal trajectories. This analysis showed three dominant patterns corresponding to early adaptive responses, stromal remodeling, and epidermal barrier maturation (Figure 2C). The early adaptive module displayed a sharp transient decline at Week 1 followed by recovery and was enriched for pathways related to structural organization and stress‐responsive signaling, including cell adhesion, junction organization, MAPK signaling, and responses to endogenous and osmotic stimuli [25, 26, 27, 28]. These changes are consistent with activation of conserved cellular stress response programs triggered by environmental perturbation and macromolecular stress [29]. In contrast, stromal remodeling programs exhibited a progressive increase over time and were enriched for response to growth factor and ECM–associated processes, including ECM regulation, collagen organization, and tissue remodeling. These signatures indicate active restructuring of stromal architecture and dynamic remodeling of matrix components during extended culture. Epidermal differentiation and barrier‐associated programs showed early enhancement at Week 1 and remained stable throughout the culture period, including epithelial differentiation, lipid localization, and homeostatic processes, reflecting progressive maturation and stabilization of the epidermal barrier.

**FIGURE 2:**
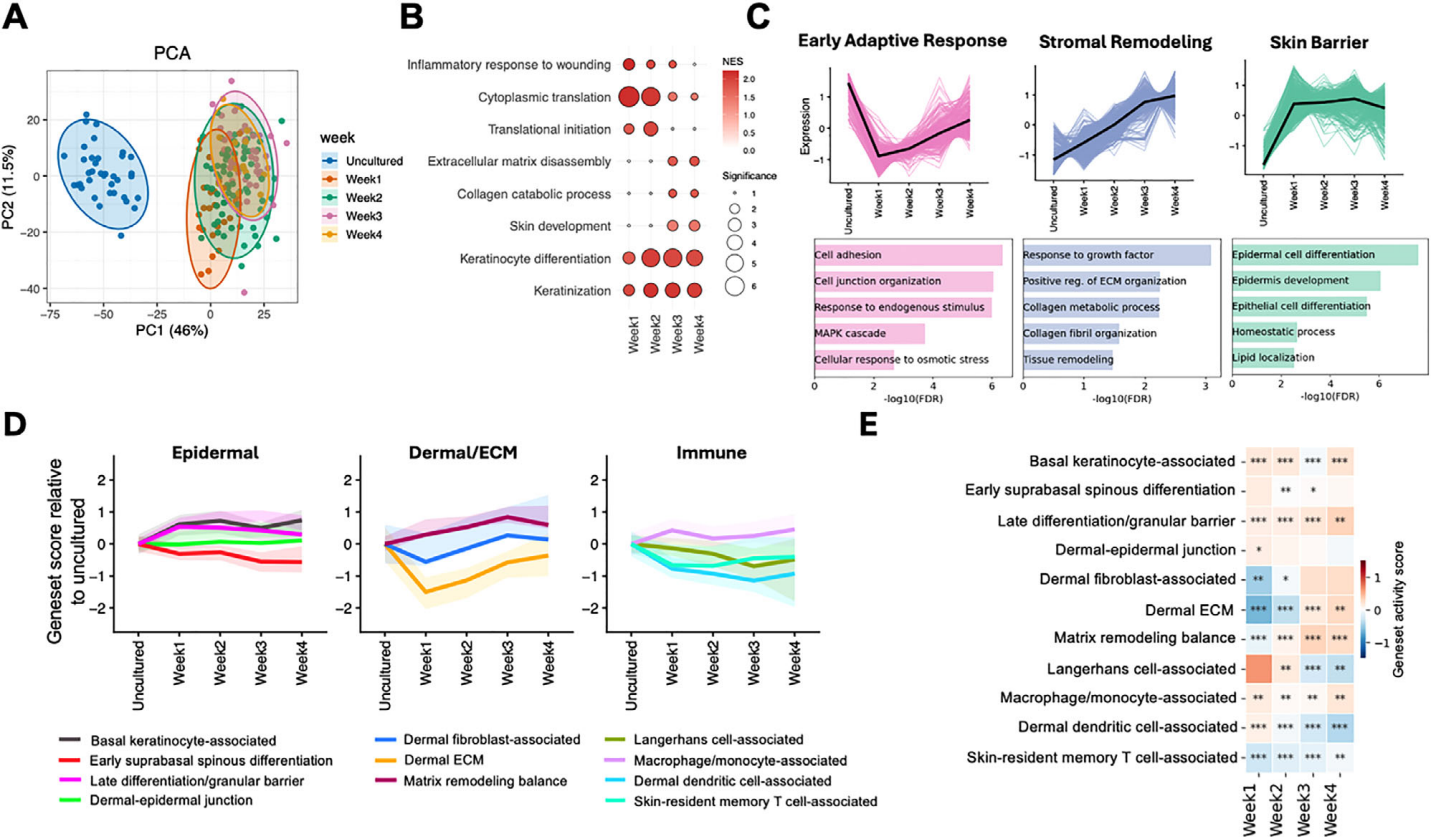
Temporal transcriptomic adaptation of human skin in the long‐term platform. (A) Principal component analysis (PCA) of uncultured skin and skin maintained in the platform for 1 to 4 weeks. Data are based on 20 unique donors (199 total tissue samples: 37, 26, 73, 48, and 15 tissue samples across uncultured and Weeks 1–4, respectively). Panels B–E were generated using samples from this longitudinal cohort. (B) Gene set enrichment analysis (GSEA) of Gene Ontology Biological Process (GOBP) terms comparing each culture week vs. uncultured tissues. Representative significantly enriched upregulated pathways (permutation‐based, preranked GSEA, BH‐adjusted FDR) are shown (FDR < 0.05; circle size indicates −log10(FDR), and color indicates normalized enrichment score (NES)). (C) Temporal gene expression trajectories of co‐expressed genes across time. Colored lines indicate individual genes, and bold black lines indicate median trajectories. Clusters reflect distinct biological programs, including early adaptive response, stromal remodeling, and skin barrier function. Corresponding enriched GOBP terms for each gene cluster are shown below each co‐expressed module (FDR < 0.05). (D) Line plots of compartment‐ and cell type‐associated transcriptional signature scores across culture time. Gene sets are grouped as epidermal, dermal/ECM, and immune‐associated programs. For each gene set and time point, plotted values represent the median gene set score difference relative to uncultured tissue. Shaded regions indicate 95% bootstrap confidence intervals (1,000 iterations). (E) Heatmap showing relative activity scores for the indicated transcriptional signatures. Asterisks indicate significant differences between each culture week and the uncultured condition using two‐sided, unpaired Welch's t‐tests with BH correction for multiple testing. ^*^ *p* < 0.05, ^**^ *p* < 0.01, ^***^ *p* < 0.001.

To further contextualize these dynamics at a compartmental level, we next examined compartment‐ and cell type‐associated transcriptional signatures using in‐house curated gene sets (Figure 2D and Table S1) [30, 31, 32, 33, 34, 35, 36, 37]. Because these signatures were derived from bulk RNA‐seq, they were interpreted as relative transcriptional activity of curated marker‐gene programs rather than direct measurements of cell abundance, proportion, or persistence. In Figure 2D, each signature was anchored to its uncultured baseline to emphasize relative temporal trajectories. Epidermal programs, including basal keratinocyte and late differentiation/barrier‐associated signatures, increased over time, whereas early suprabasal differentiation signatures exhibited a modest reduction, indicating layer‐specific modulation with overall maintenance of epidermal differentiation and maturation. Stromal signatures, including dermal ECM and fibroblast‐associated programs, displayed an early decrease followed by recovery over time, accompanied by progressive enrichment of matrix remodeling signatures, consistent with dynamic ECM modulation. Immune‐associated signatures showed more modest and heterogeneous temporal shifts across culture time points, with distinct patterns observed among different immune‐associated transcriptional programs. To complement these trajectory plots, Figure 2E displays gene set activity scores in heatmap form, facilitating visualization of gene sets with concordant temporal patterns across culture time points. This representation highlights sustained epidermal differentiation/barrier activity, dynamic matrix remodeling activity, and heterogeneous immune‐associated transcriptional patterns. Together, these results demonstrate coordinated but non‐uniform regulation of epidermal, stromal, and immune‐associated transcriptional programs over time. These changes reflect ongoing remodeling and a homeostasis‐like balance rather than progressive deterioration, consistent with preservation of tissue architecture observed histologically (Figure 1).

### 2.3 Long‐Term Cytokine Stimulation Induces Sustained Inflammatory Remodeling that is Attenuated by Tofacitinib

To define the effects of prolonged inflammatory stimulation, we performed longitudinal transcriptomic, histological, and protein‐level analyses of IL‐17/IL‐22‐treated human skin tissues over a 3‐week period, with or without tofacitinib, a Janus kinase (JAK) inhibitor that suppresses cytokine‐dependent inflammatory signaling and has demonstrated efficacy in immune‐mediated diseases such as psoriasis [38] (Figure 3A). At Week 1 of treatment, IL‐17/IL‐22 induced a robust transcriptional response characterized by strong upregulation of inflammatory mediators, including *IL36A, DEFB4A/B, S100A12, CXCL1, CXCL8, IL6*, and *MMP1*, alongside suppression of epidermal differentiation and barrier genes such as *FLG, FLG2, LORICRIN, KRT77*, and *DSC1* (Figure 3B). These changes are consistent with cytokine‐driven inflammatory activation and impaired barrier function, reflecting established IL‐17‐driven inflammatory activation and IL‐22–associated suppression of keratinocyte differentiation [39, 40, 41]. GSEA of the top enriched GOBP terms further revealed enrichment of immune‐related pathways, including leukocyte chemotaxis and antimicrobial responses, coupled with suppression of pathways linked to epidermal differentiation and lipid metabolism (Figure 3C). At the pathway level, this profile closely parallels psoriasis‐relevant inflammatory transcriptional signatures, characterized by coordinated induction of inflammatory programs and disruption of epidermal differentiation and barrier‐associated pathways [39, 41]. Consistent with these findings, hallmark pathway analysis demonstrated sustained activation of inflammatory signaling programs, including TNFα/NF‐κB, IL6/JAK/STAT3, and inflammatory response pathways, which were attenuated by tofacitinib [38, 42] (Figure 3D). These findings further support sustained tissue‐level inflammatory responsiveness over extended culture durations, enabling recapitulation of key inflammatory signaling events in human skin.

**FIGURE 3:**
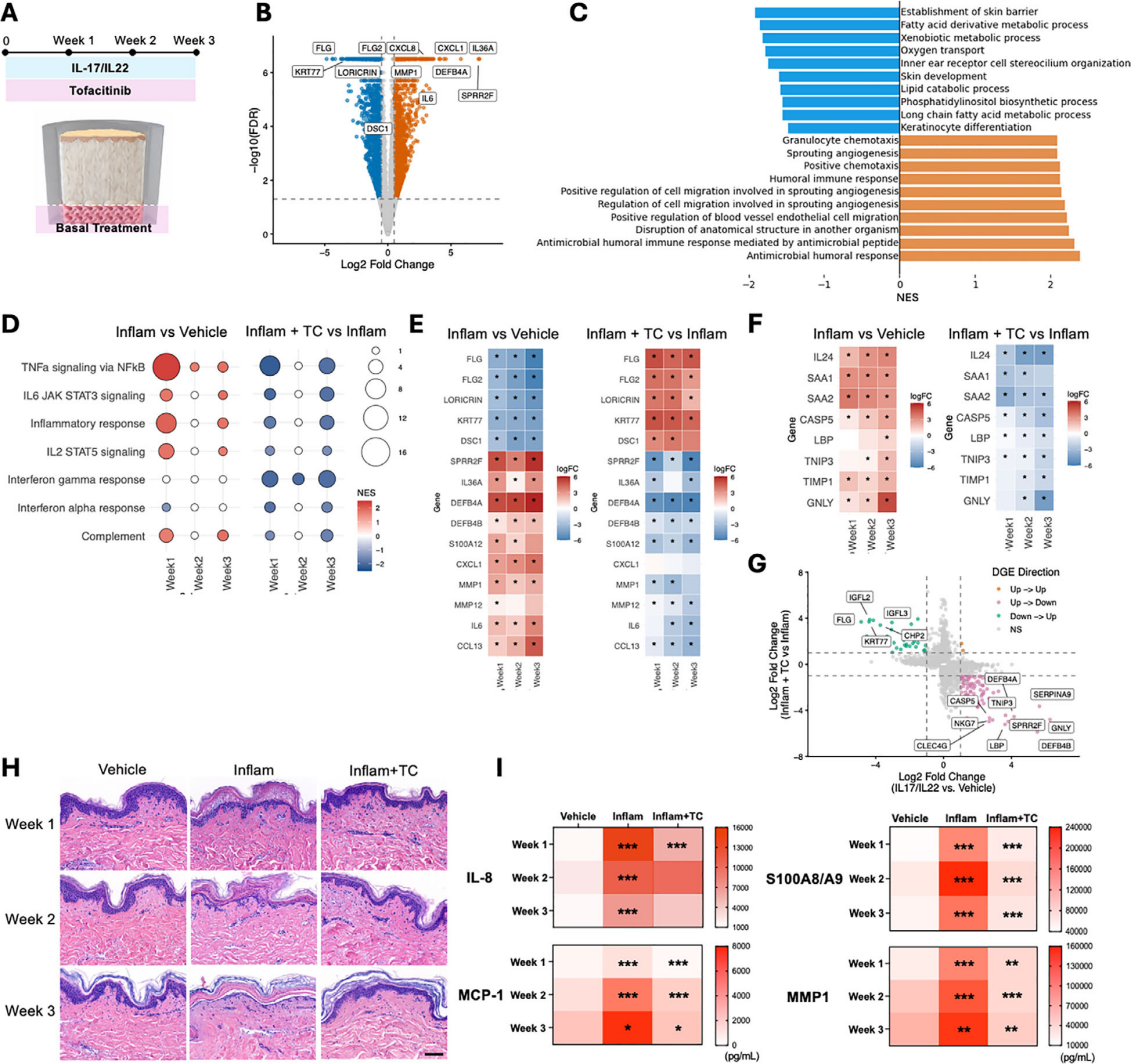
Longitudinal cytokine‐driven inflammation and tofacitinib intervention. (A) Schematic of experimental design showing IL‐17/IL‐22 (Inflam) stimulation with or without tofacitinib (TC) over 3 weeks following 2–3 days of initial culture (23–24 days total culture). (B) Volcano plot of differential gene expression at Week 1 comparing inflamed vs. vehicle‐treated tissues. Selected psoriasis‐associated marker genes are indicated. Genes passing significance thresholds (DESeq2, two‐sided Wald test, FDR < 0.05, |log2 fold change| >0.5) are colored by direction of change: blue, downregulated; orange, upregulated. *N* = 3 unique donors, 24 tissue samples total. (C) GSEA of GOBP terms comparing inflamed vs. vehicle‐treated tissues. The top significantly enriched upregulated and downregulated pathways are shown (permutation‐based, preranked GSEA, BH‐adjusted FDR < 0.05, *n* = 3 donors, consistent with panel B). (D) GSEA of Hallmark pathways comparing inflamed vs. vehicle and inflamed + TC vs. inflamed across time points (permutation‐based, preranked GSEA, BH‐adjusted FDR < 0.05, *n* = 3 donors, 72 tissue samples total). Circle size indicates −log10(FDR), and color indicates NES. (E) Heatmap of selected differentially expressed genes across vehicle, inflamed, and inflamed + TC over time (DESeq2, two‐sided Wald test, FDR < 0.05). Values represent weekly log2 fold changes relative to the vehicle (left heatmap) or inflamed condition (right heatmap). *N* = 3 donors, 72 tissue samples total. (F) Heatmap of additional chronic inflammation‐associated genes across the 3‐week time course (DESeq2, two‐sided Wald test, FDR < 0.05). Values represent weekly log2 fold changes relative to the vehicle (left heatmap) or inflamed condition (right heatmap). Asterisks indicate a significant difference in gene expression compared to the control group at FDR < 0.05. *N* = 3 donors, 72 tissue samples total. (G) Transcriptomic reversal plot at Week 3 showing the relationship between gene expression changes induced by inflammation (vs. vehicle) and changes upon TC treatment (inflamed + TC vs. inflamed), based on differentially expressed genes (DESeq2, two‐sided Wald test, BH‐adjusted FDR < 0.05). *N* = 3 donors, 44 tissue samples total. (H) Representative H&E staining of vehicle, inflamed, and inflamed + TC‐treated tissues at Weeks 1, 2, and 3. Scale bar, 100 µm. (I) Heatmap of secreted protein levels (IL‐8, MCP‐1, S100A8/A9, and MMP‐1) across treatment groups and time points. The heatmap represents mean secreted protein concentrations aggregated across replicate measurements from multi‐donor experiments and visualized using a single gradient color scale. Data are presented from 3–4 donors (Weeks 1 and 2: 4 donors; Week 3: 3 donors; *n* = 3–9 technical replicates per group per donor per time point). Statistical significance was assessed using a mixed‐effects model (REML) with Geisser–Greenhouse correction, followed by Dunnett's multiple‐comparisons test within each time point. Asterisks indicate comparisons between inflamed vs. vehicle and inflamed + TC vs. inflamed groups. ^*^ *p* < 0.05, ^**^ *p* < 0.01, ^***^ *p* < 0.001.

To assess temporal dynamics, we tracked representative inflammatory and barrier‐associated genes identified in Figure 3B across the 3‐week treatment period (Figure 3E). These genes remained consistently regulated over time, with persistent suppression of barrier‐associated genes and sustained induction of inflammatory and remodeling‐associated genes across all three weeks. Tofacitinib effectively attenuated both inflammatory gene induction and barrier gene repression across all time points, consistent with inhibition of JAK‐dependent signaling components of the cytokine response, particularly IL‐22‐associated signaling, with secondary suppression of the broader inflammatory program. The sustained responsiveness to tofacitinib further indicates that immune signaling pathways remain active and pharmacologically responsive in the platform over the 3‐week period. Prolonged cytokine exposure sustained a set of inflammatory and stress‐associated transcripts across the 3‐week period, including *IL24, SAA1/2, CASP5, LBP, TNIP3, TIMP1*, and *GNLY* [43, 44, 45, 46] (Figure 3F). These changes were consistently attenuated by tofacitinib, indicating suppression of both early and sustained components of the inflammatory response. Transcriptomic reversal analysis further demonstrated a shift of IL‐17/IL‐22‐induced genes toward baseline expression following tofacitinib treatment (Figure 3G).

Histological analysis supported these transcriptional findings (Figure 3H). Untreated (data not shown) and vehicle‐treated tissues maintained overall epidermal and dermal architecture across all time points. In contrast, IL‐17/IL‐22‐treated tissues exhibited progressive epidermal alterations, including dyskeratosis, basal vacuolar changes, and intracellular edema at Week 1, progressing to parakeratosis and subcorneal separation by Weeks 2 and 3. By Week 3, prominent sloughing of the stratum corneum indicated advanced epidermal barrier disruption. Tofacitinib attenuated these changes, with tissue architecture remaining comparable to vehicle controls and showing minimal epidermal changes. Consistent with these observations, IL‐17/IL‐22 stimulation induced sustained secretion of IL‐8, MCP‐1, S100A8/A9, and MMP‐1 across all time points. Tofacitinib broadly attenuated this inflammatory protein response, with significant reductions in MCP‐1, S100A8/A9, and MMP‐1 across time points and more modest attenuation of IL‐8, consistent with selective inhibition of JAK‐dependent cytokine signaling and partial persistence of JAK‐independent inflammatory pathways [38, 47] (Figure 3I). Collectively, these data demonstrate that the platform faithfully recapitulates key features of cytokine‐driven inflammatory responses observed in human skin, while enabling longitudinal assessment of pharmacologic modulation at transcriptional, structural, and functional levels.

### 2.4 A Long‐Term Human Skin Platform Enables Evaluation of Delayed Senolytic‐Associated Responses in Aged Donor Skin

Because senolytic‐associated effects often emerge over extended timeframes and are difficult to capture in short‐term in vitro systems, we next asked whether the platform could elucidate delayed treatment‐associated responses in aged tissue. To this end, skin tissues from aged donors (58–74 years) were maintained for three weeks with repeated exposure in the culture medium to vehicle, dasatinib, a tyrosine kinase inhibitor with senolytic activity, or dasatinib plus quercetin (D+Q), a senolytic‐associated combination commonly used in aging studies [48, 49] (Figure 4A). Analyses at earlier time points showed minimal transcriptional and protein‐level responses (Figure S2), consistent with an extended response window over which senolytic‐associated effects have been reported to emerge in vivo despite the short pharmacokinetic half‐lives of these agents [49, 50, 51]. Histological assessment at Week 3 showed preservation of overall epidermal and dermal architecture across all treatment groups, with no overt evidence of tissue disruption following prolonged treatment (Figure 4B). At the protein level, both dasatinib and D+Q reduced IL‐6 and MMP‐1 relative to vehicle at Week 3 (Figure 4C).

**FIGURE 4:**
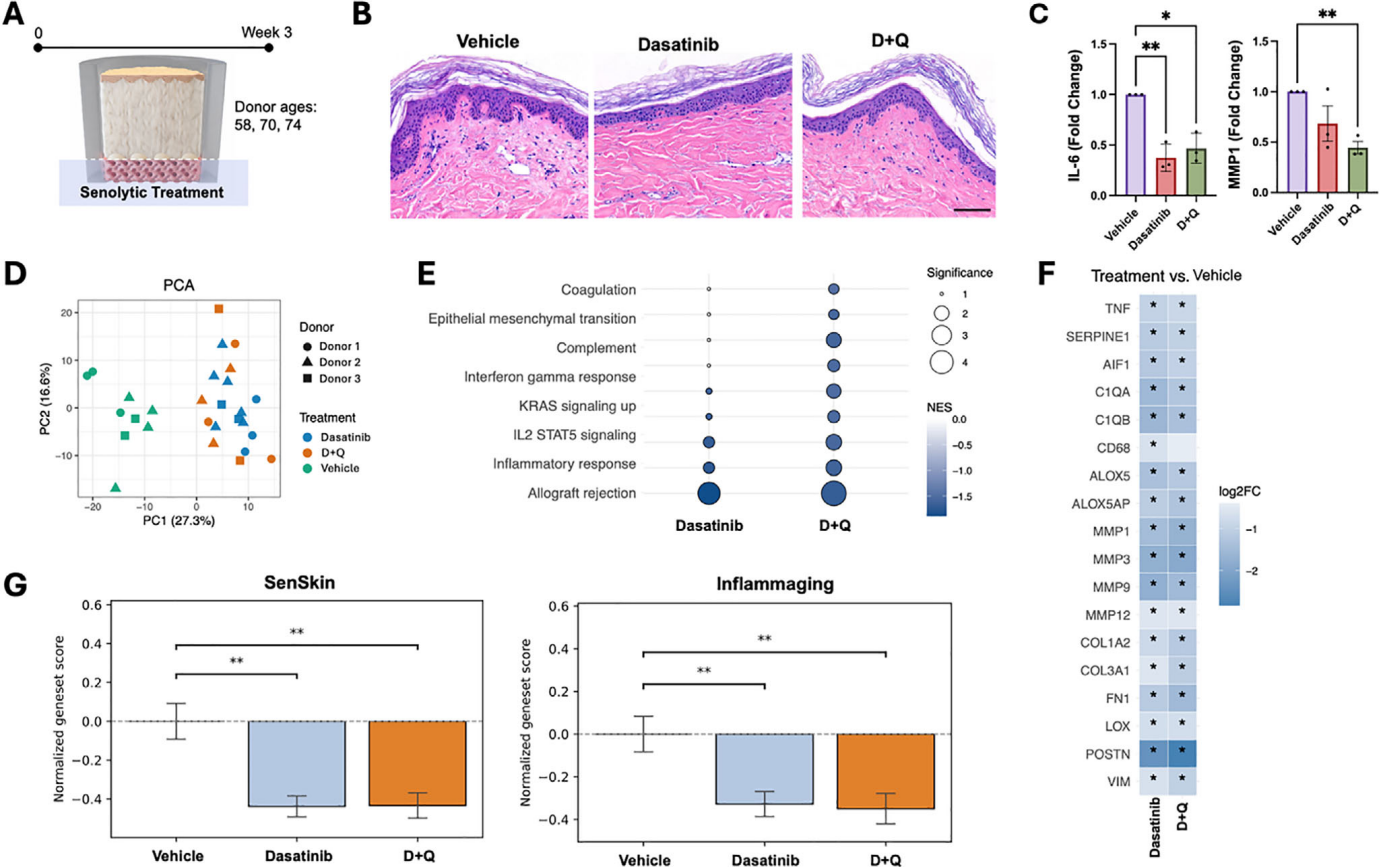
Long‐term senolytic treatment in aged skin. (A) Schematic of the experimental design for treatment of aged human skin with dasatinib or dasatinib plus quercetin (D+Q) for 3 weeks following 3–5 days of initial culture (24–26 days total culture). Donor ages are indicated. (B) Representative H&E staining of vehicle‐, dasatinib‐, and D+Q‐treated tissues. Scale bar, 100 µm. (C) Quantification of IL‐6 and MMP‐1 at Week 3 (fold change relative to vehicle control). Data are presented as mean ± SEM from 3 donors (*n* = 3–5 technical replicates per group per donor). Technical replicates were averaged within each donor and treatment group before statistical analysis. Statistical significance was assessed using one‐way repeated‐measures ANOVA followed by Dunnett's multiple‐comparisons test versus vehicle. ^*^ *p* < 0.05, ^**^ *p* < 0.01, ^***^ *p* < 0.001. (D) Principal component analysis (PCA) plot of transcriptomic profiles stratified by donor and treatment group. *N* = 3 donors, 28 tissue samples total. (E) Selected pathways from GSEA of Hallmark pathways comparing treatment vs. vehicle (permutation‐based, preranked GSEA, BH‐adjusted FDR < 0.05). Circle size indicates −log10(FDR), and color indicates NES. *N* = 3 donors, consistent with panel D. (F) Heatmap of differentially expressed genes associated with inflammation and matrix remodeling comparing treatment to vehicle (DESeq2, two‐sided Wald test, BH‐adjusted FDR). Values represent log2 fold changes relative to vehicle. Asterisks indicate a significant difference in gene expression compared to the vehicle group at FDR < 0.05. (G) Gene set scores for SenSkin and custom inflammaging signatures across treatment groups relative to vehicle. Statistical significance was assessed using a two‐sided, unpaired Welch's t‐test with BH correction for multiple testing. Data are presented as median ± SEM from 3 donors (*n* = 2–6 technical replicates per group).

PCA of bulk RNA‐seq data revealed separation between vehicle and senolytic‐treated samples along the primary principal component, while dasatinib and D+Q largely overlapped, indicating similar transcriptional effects (Figure 4D). GSEA showed coordinated downregulation of immune and inflammatory programs, including IL2–STAT5 signaling, interferon gamma response, and inflammatory response, together with suppression of epithelial–mesenchymal transition and related remodeling‐associated pathways (Figure 4E). Gene‐level analysis supported this pattern, with reduced expression of canonical SASP‐associated, inflammatory, and matrix remodeling‐related genes (Figure 4F). *ALOX5* and *ALOX5AP*, components of lipid mediator synthesis pathways, were also reduced following treatment [52]. Genes involved in ECM organization and remodeling were modestly downregulated together with matrix‐degrading enzymes, consistent with attenuation of active tissue remodeling.

To assess aging‐associated transcriptional states, we quantified a published senescence–associated skin gene signature (SenSkin) [53] and an in‐house curated inflammaging signature (Table S2). Both dasatinib and D+Q significantly reduced SenSkin and inflammaging scores relative to vehicle (Figure 4G), with broadly similar effects between the two treatments. Together, these findings demonstrate that the platform enables detection of prolonged senolytic‐associated transcriptional responses in human tissue, providing a unique framework to evaluate therapeutic effects that emerge over extended timescales and are difficult to study in short‐term in vitro systems and conventional experimental models of human aging.

### 2.5 Functional Barrier Competence for Topical Intervention: UVB Induces Epidermal Injury Responses and Inflammatory Remodeling

To model UV‐induced skin damage and evaluate the platform's capacity for real‐world topical application paradigms–a common limitation of in vitro models with altered permeability–human skin tissues were first stabilized in culture for 3–5 days and then subjected to a single ultraviolet B (UVB) exposure (300 mJ/cm^2^) [54, 55] in the presence or absence of topically applied sunscreen, followed by an additional 3 days of culture (Figure 5A). Histological analysis revealed that UVB exposure induced marked epidermal and dermal pathology, including diffuse parakeratosis, dyskeratosis, and hyperkeratosis in the epidermis, as well as focal lymphohistiocytic infiltration and collagen bundle fragmentation in the dermis compared with untreated controls (Figure 5B). These morphological changes were substantially mitigated by sunscreen treatment, which preserved overall tissue structure. Consistent with tissue injury, UVB exposure significantly increased lactate dehydrogenase (LDH) release, indicating cytotoxicity, and elevated secretion of the pro‐inflammatory cytokine IL‐8 in the basal medium (Figure 5C). Both responses were significantly reduced by sunscreen treatment, suggesting attenuation of UV‐induced tissue injury and inflammatory signaling. Sunscreen treatment alone did not induce detectable histological or biochemical changes compared to untreated controls (data not shown).

**FIGURE 5:**
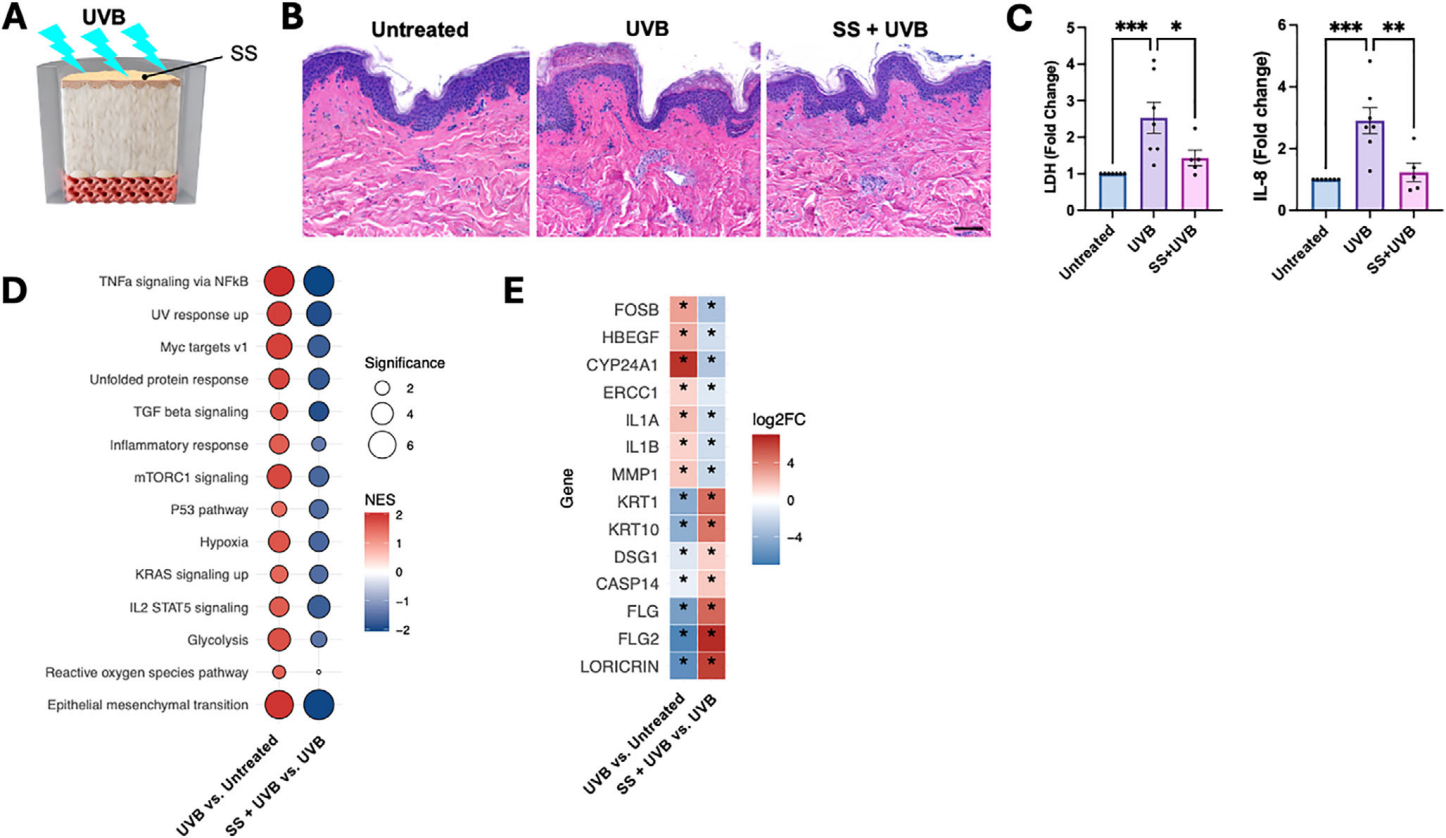
UVB‐induced responses and sunscreen protection in the human skin platform. (A) Schematic of the experimental design for UVB exposure (300 mJ/cm^2^) with or without sunscreen (SS). (B) Representative H&E staining of untreated, UVB, and SS + UVB‐treated tissues collected 3 days after UVB exposure. Scale bar, 100 µm. (C) Quantification of LDH release and IL‐8 secretion in basal medium collected on Day 3 after UVB exposure across treatment groups (fold change relative to untreated control). Data are presented as mean ± SEM from 5–7 donors (*n* = 3–5 technical replicates per group per donor). Technical replicates were averaged within each donor and treatment group before statistical analysis. Statistical significance was evaluated by one‐way repeated‐measures ANOVA followed by Dunnett's multiple‐comparisons test, with the UVB group as the reference. Asterisks indicate comparisons of UVB vs. untreated and SS + UVB vs. UVB; ^*^ *p* < 0.05, ^**^ *p* < 0.01, ^***^ *p* < 0.001. (D) Selected pathways from GSEA of Hallmark pathways comparing UVB vs. untreated and SS + UVB vs. UVB (permutation‐based, preranked GSEA, BH‐adjusted FDR < 0.05). Circle size indicates −log10(FDR), and color indicates NES. *N* = 3 donors, 36 tissue samples total. (E) Heatmap of selected genes showing transcriptional responses to UVB exposure and sunscreen treatment (DESeq2, two‐sided Wald test, BH‐adjusted FDR). Values represent log2 fold changes relative to the control indicated on the x‐axis labels. Asterisks indicate significant differences in gene expression for the indicated comparison at FDR < 0.05. *N* = 3 donors, 36 tissue samples total.

To elucidate the molecular programs underlying these responses, we performed transcriptomic profiling followed by GSEA. UVB exposure robustly activated pathways associated with inflammatory signaling, cellular stress, and tissue remodeling, including TNFα signaling via NF‐κB, inflammatory response, UV response, and unfolded protein response (Figure 5D). Additional enrichment of MYC, mTORC1, KRAS, and hypoxia pathways indicated broader metabolic and stress‐adaptive responses, consistent with in vivo UV exposure studies demonstrating temporally structured transcriptional programs, as well as well‐established UV‐induced inflammatory signaling mediated by reactive oxygen species (ROS), NF‐κB, and pro‐inflammatory cytokines [56, 57]. These UV‐induced pathway signatures were broadly attenuated in sunscreen‐treated samples, indicating suppression of stress and inflammatory programs. At the gene level, UVB exposure induced a coordinated transcriptional response involving cellular stress, inflammatory signaling, tissue remodeling, and epidermal differentiation (Figure 5E). UV‐responsive genes, including *FOSB, HBEGF, CYP24A1, ERCC1, IL1A, IL1B*, and *MMP1*, were significantly induced following UVB exposure and attenuated by sunscreen treatment. Conversely, multiple epidermal differentiation and barrier‐associated genes, including *KRT1, KRT10, DSG1, CASP14, FLG, FLG2*, and *LORICRIN*, were significantly suppressed by UVB and restored toward baseline with sunscreen treatment (Figure 5E). The ability of the platform to support and evaluate this topical formulation without non‐specific toxicity or barrier failure demonstrates its practical compatibility with topical treatment under preserved tissue architecture. Together, these results demonstrate that the platform recapitulates coordinated UV‐induced inflammatory and tissue injury responses consistent with in vivo human skin, while enabling quantitative evaluation of topical intervention efficacy within a preserved tissue architecture.

## 3 Discussion

Persistent inflammatory signaling contributes to tissue dysfunction across both disease‐associated and aging‐associated contexts [6, 7], and these processes can be further amplified by environmental stressors [16, 17]. However, resolving how these diverse inputs converge to drive structural tissue deterioration has been limited by the lack of human‐relevant models capable of capturing these processes over extended timeframes. Here, we establish a long‐term, immune‐responsive human skin platform that enables longitudinal interrogation of these dynamics within an intact tissue context. By preserving tissue architecture and supporting sustained immune–stromal signaling for up to 4 weeks, the system maintains key aspects of tissue functionality and provides a tractable framework to investigate chronic tissue stress and aging‐associated processes with temporal resolution.

Although extended ex vivo skin culture has been reported, progressive culture‐associated changes can limit tissue integrity and experimental utility over time [20, 21, 58]. The platform presented here combines a custom 3D‐printed tissue insert with a highly porous (∼70%) open architecture designed to facilitate culture medium access and nutrient/waste exchange while maintaining a stable ALI. This configuration supports reproducible, multi‐week maintenance of human skin architecture in a scalable 24‐well plate format. Consistent with this design, comparison with a conventional transwell‐based configuration showed comparable overall tissue architecture at Day 7 but clear divergence by Day 15, with greater preservation of epidermal–dermal architecture in the 3D‐printed insert platform (Figure S1). Longitudinal transcriptomic analysis further revealed that, following an early adaptation phase, tissues stabilize into a differentiated and transcriptionally consistent state rather than undergoing progressive deterioration. The current study was not designed to isolate the individual contribution of each engineering parameter to extended tissue maintenance. Although the system does not recapitulate recruitment of circulating immune cells, immune‐associated and stromal transcriptional programs remained detectable over extended culture durations, while prolonged cytokine stimulation demonstrated sustained tissue‐level inflammatory responsiveness and pharmacological modulation. Together, these features bridge a key gap between the native complexity of ex vivo human skin and the extended experimental accessibility of simplified in vitro systems. Histological evidence of lymphohistiocytic infiltration in the UV study further supports the presence and local responsiveness of resident immune components within the ex vivo platform.

A central insight from this model is that distinct biological stressors—including chronic immune activation, senescence‐associated signaling, and environmental damage—elicit overlapping features of inflammatory activation, ECM remodeling, and epidermal disruption. These responses were characterized by induction of matrix metalloproteinases, altered keratinocyte differentiation programs, and persistent cytokine signaling, consistent with mechanisms implicated in age‐associated tissue dysfunction. Rather than representing independent processes, these findings suggest that diverse stress inputs engage overlapping inflammatory and tissue‐remodeling programs, providing a framework for understanding how distinct drivers of tissue stress may contribute to progressive structural decline.

The extended culture duration of the platform enables resolution of distinct temporal kinetics across different stress modalities. Acute environmental insults such as UV exposure induce rapid tissue injury that can be captured over short timeframes, whereas chronic immune activation and aging‐associated processes develop over prolonged periods. Cytokine‐driven inflammation established a sustained yet pharmacologically reversible state over weeks, while senescence‐targeting interventions in aged tissues produced delayed but coordinated attenuation of inflammatory and remodeling‐associated transcriptional programs. These delayed responses are consistent with the gradual kinetics reported in vivo, highlighting the importance of extended observation windows for evaluating therapies targeting chronic tissue states [49, 50, 51].

From a translational perspective, this platform addresses a key gap between conventional in vitro systems and in vivo human skin biology. Conventional models often fail to capture coordinated interactions between epithelial, stromal, and immune compartments that govern tissue‐level responses. By maintaining these interactions within a controlled environment, the system enables integrated assessment of disease‐relevant perturbations and therapeutic interventions at molecular, structural, and functional levels. In addition, preservation of stratified epidermal architecture enables evaluation of topically applied interventions within a physiologically relevant barrier context. This is particularly important for dermatologic interventions, for which clinical translation is often constrained by skin penetration, formulation stability, and limited mechanistic validation [59]. The ability to assess UV‐induced damage and its modulation by sunscreen therefore demonstrates the platform's practical compatibility with topical treatment paradigms that remain challenging to model in conventional in vitro systems [60, 61].

Several limitations should be considered. Although resident immune cell‐associated transcriptional programs remained detectable and tissues retained inflammatory responsiveness during extended culture, bulk RNA‐seq does not directly quantify the abundance or persistence of specific resident immune cell populations. More broadly, the current ex vivo platform does not recapitulate the recruitment of circulating immune cells or systemic immune interactions. The absence of systemic vascular and lymphatic circulation precludes recruitment of circulating leukocytes, limiting the modeling of dynamic immune trafficking and perfusion‐dependent tissue responses. Future incorporation of circulating immune cell components may enable modeling of more complex immune responses. Although histological and transcriptomic analyses support preservation of tissue architecture and molecular programs over the validated culture window, viability and metabolic activity of all cell populations throughout the full thickness of the explant were not directly assessed. Finally, although the use of primary human explants introduces donor‐to‐donor variability, the cohort included diverse donor characteristics, enabling capture of biologically relevant heterogeneity. The present study was not designed to evaluate responses stratified by donor demographics.

In summary, this long‐term human skin platform provides a versatile system for modeling chronic inflammatory and aging‐associated tissue dysfunction. By enabling investigation of chronic inflammatory signaling, senescence‐associated remodeling, and environmental stress within a unified and temporally resolved framework, the model offers a powerful approach for studying mechanisms of tissue aging and evaluating therapeutic strategies targeting a wide range of skin conditions.

## 4 Conclusion

We establish a long‐term ex vivo human skin platform that enables multi‐week maintenance of native tissue architecture, multicellular tissue context, and molecular fidelity, supporting longitudinal interrogation of complex tissue dynamics. The system captures key dimensions of chronic tissue stress, including sustained cytokine‐driven inflammation, senescence‐associated remodeling in aged donor tissue, and UV‐induced environmental injury, each eliciting coordinated inflammatory and structural responses that can be modulated pharmacologically or through topical intervention. This platform bridges a critical gap between in vitro systems and in vivo human skin biology in terms of evaluation duration and human‐relevant biological complexity. As such, it provides a versatile and translationally relevant framework for investigating mechanisms of tissue dysfunction and for evaluating therapeutic strategies targeting chronic inflammatory and aging‐associated processes.

## 5 Methods

### 5.1 Insert Manufacturing

Skin biopsy support structures were modeled using Fusion360 (Version 2.0, Autodesk, San Rafael, CA, USA), sliced with PreForm (Version 3.40), and printed on a Formlabs 4B+ printer using Biomed Clear resin (Formlabs Inc., Somerville, MA, USA) at a 50 µm layer height. Parts were washed in 99% isopropyl alcohol for 20 min, dried with compressed air, cured for 60 min in a Formlabs Cure UV oven at 60°C, and autoclaved before use.

### 5.2 Tissue Preparation

Fresh de‐identified human skin tissue from healthy donors was procured from both commercial and non‐profit suppliers of tissue for research in compliance with collection site institutional review board (IRB) protocols and applicable state and federal ethical regulations. Fresh skin tissues, typically measuring approximately 100 cm^2^ in size, were received within 24 h of surgical removal under controlled conditions. Upon receipt, the tissues were processed under sterile conditions. Full‐thickness skin biopsies (8 mm diameter) were generated using custom cutting dies and a pneumatic press (Tippmann, USA). Each sample was trimmed to remove excess subcutaneous fat and underwent a final quality assessment prior to experimental use.

### 5.3 Tissue Culture

Across all studies, tissues were obtained from donors spanning 19–74 years of age and included both female and male donors, with Fitzpatrick phototypes I–V represented. All biopsies were transferred and cultured in sterilized, custom‐built 3D‐printed insert structures designed to maintain an ALI and enhance nutrient transport. Each biopsy was housed in an individual insert designed to fit in 24‐well plates (VWR, Radnor, PA, USA) and supplied with 700 µL of culture medium in the basal compartment. The culture medium consisted of a defined formulation based on Williams’ E medium (Gibco, A12176‐01, Thermo Fisher Scientific, Waltham, MA, USA) supplemented with GlutaMAX (Gibco), non‐essential amino acids (Gibco), ITS (Corning), Amphotericin B and penicillin‐streptomycin (Thermo Fisher Scientific). These cultures were maintained at 37°C, 5% CO_2_, and 95% humidity. Culture medium was replaced every 1–2 days, and effluent was collected for biochemical assays. No separate keratinocyte differentiation medium was used, and no exogenous growth factors were added to the maintenance medium used during the extended culture and treatment periods. For comparison with a conventional culture format, biopsies from the same donor were cultured in parallel using Corning Transwell permeable supports (0.4 µm pore size, polycarbonate membrane; Corning, 3401) using the same culture medium and incubation conditions and harvested on Days 7 and 15 for histological analysis.

### 5.4 Inflammation Induction and Treatments

Skin tissue samples from four donors (Donor 1, 36‐year‐old female; Donor 2, 32‐year‐old female; Donor 3, 19‐year‐old female; Donor 4, 52‐year‐old female) were stabilized in culture for 2–3 days prior to treatment. Tissues were then subjected to inflammatory stimulation using a cytokine cocktail. Biopsies assigned to the inflammation group (Inflam) were treated via the basal medium every other day with recombinant human IL‐17A (50 ng/mL, BioTechne, 7955‐IL‐025, Minneapolis, MN, USA) and recombinant human IL‐22 (10 ng/mL, BioTechne, 782‐IL‐010). An anti‐inflammatory agent, tofacitinib citrate (TC, 5 µM; MedChemExpress (MCE), HY‐40354A, Monmouth Junction, NJ, USA), was administered concurrently with the Inflam group cytokines. Treatments were continued longitudinally for 3 weeks (21 days). Effluents were collected and replaced with fresh treatment medium every other day throughout the treatment period, with effluent analyses performed on samples collected at Weeks 1, 2, and 3. Tissues were harvested at the corresponding time points for downstream analyses.

### 5.5 Senolytic Treatment

Skin tissue samples from three donors (Donor 1, 74‐year‐old female; Donor 2, 58‐year‐old male; Donor 3, 70‐year‐old male) were stabilized in culture for approximately 3–5 days prior to treatment. Tissues were then treated via the basal medium with senolytic agents to evaluate prolonged treatment‐associated responses in aged skin tissue. Treatment groups included dasatinib (50 nM, MCE, HY‐10181) and a combination of dasatinib (50 nM) and quercetin (10 µM, MCE, HY‐18085). Effluents were collected every other day and replaced with fresh medium throughout the 21‐day treatment period, and tissues were harvested at the corresponding time points for downstream analyses.

### 5.6 UV Treatment

Skin tissue samples from seven donors (Donor 1, 38‐year‐old female; Donor 2, 33‐year‐old female; Donor 3, 21‐year‐old female; Donor 4, 60‐year‐old female; Donor 5, 36‐year‐old female; Donor 6, 25‐year‐old female; Donor 7, 53‐year‐old female) were maintained in culture for an initial stabilization period of 3–5 days prior to UVB exposure. Tissues were then exposed to UVB radiation using a UV crosslinker (306 nm; Model 234100, Boekel Scientific, Feasterville, PA, USA). For the irradiation procedure, biopsies from tissue culture plates were transferred to sterile Petri dishes (Greiner Bio‐One, 07‐000‐335, Monroe, NC, USA) containing sterile gauze pads pre‐soaked in sterile Dulbecco's phosphate‐buffered saline (D‐PBS; Sigma–Aldrich, 56064C, St. Louis, MO, USA) to maintain tissue hydration. These Petri dishes were placed at a fixed distance from the UV source to ensure uniform exposure. Samples received a single UVB exposure of 300 mJ/cm^2^ UVB. For the sunscreen treatment group, skin biopsies were pre‐treated 15 min prior to UV exposure with a commercially available sunscreen (Neutrogena, SPF 50). A volume of 2 µL (∼4 µL/cm^2^) was applied to the epidermal surface using a positive displacement pipette (Gilson, Middleton, WI, USA) and was evenly spread using a sterile glass rod. The control samples were handled identically but maintained in culture in the biosafety cabinet for the same duration without UV exposure. Following exposure, samples were returned to the culture platform with fresh medium and incubated for an additional 3 days at 37°C, 5% CO_2_, and 95% humidity. Effluents were collected and replaced with fresh culture medium daily, and tissues were harvested 3 days after UVB exposure for downstream analyses.

### 5.7 Tissue Harvest and Sample Processing

At each designated tissue harvest, biopsies intended for paired histological and transcriptomic analyses were bisected perpendicular to the epidermal surface, from the epidermis through the dermis, using a sterile surgical blade (Havalon, 70A, Cincinnati, OH, USA). One half was fixed in 10% formalin for histological analysis, and the other half was placed in RNAlater (Sigma‐Aldrich, R0901) and stored at −80°C for RNA‐seq analysis.

### 5.8 RNA‐seq Processing and Quantification

Samples designated for RNA‐seq were shipped on dry ice to Azenta Life Sciences (South Plainfield, NJ, USA) for RNA extraction, library preparation, and next‐generation sequencing. Total RNA was extracted using the Qiagen RNeasy Plus Universal Mini kit following manufacturer's instructions (Qiagen, Hilden, Germany). Libraries were prepared and sequenced on a NovaSeq (Illumina, San Diego, CA, USA) with 2 × 150 bp paired‐end reads (∼20 M reads/sample). Data were processed using nf‐core/rnaseq v3.19.0 [62] of the nf‐core collection of workflows [63] with default parameters. The pipeline was executed with Nextflow v25.10.4 [64]. Gene annotations were based on GENCODE v38. Quality control metrics generated by the nf‐core/rnaseq pipeline were evaluated for low‐quality samples. Evaluated metrics included rRNA percentage, number and percentage of aligned reads, deduplication rate, and GC content. No samples were excluded based on QC metrics. Principal component analysis (PCA) was used to assess sample clustering and identify potential outliers. One sample was considered an outlier as it clustered distinctly from other replicates in the same treatment group and was excluded.

### 5.9 Differential Gene Expression (DGE) analysis

We adopted the DGE approach outlined in the nf‐core/differentialabundance pipeline (v1.5.0) [65] with minor modifications as outlined below. Count matrices were analyzed for differential expression using DESeq2 (v1.50.2) [66]. Log2 fold‐change estimates were shrunk using the adaptive shrinkage (ashr) method. Unless specified otherwise, genes were considered significantly differentially expressed at a Benjamini‐Hochberg (BH)‐adjusted false discovery rate (FDR) < 0.05. The design formula included the primary contrast of interest, with donor included as a blocking factor in analyses involving multiple donors, along with relevant covariates such as batch, culture week, and interaction terms where applicable. As Salmon was used for transcript quantification, gene length information was incorporated as an assay (avgTxLength) in the DESeqDataSet object to account for gene length differences. Batch effects were removed using the removeBatchEffect function from the limma package (v3.66.0) [67] on variance‐stabilizing transformation (VST)‐transformed data, and the corrected matrix was used for downstream analyses (referred to as “normalized data” throughout the manuscript). Specifically, Figures 2C–E, 4D,F,G, and 5E were generated based on batch‐corrected data.

### 5.10 Gene Set Enrichment Analysis (GSEA)

Genes were ranked based on the log2 fold changes output by DESeq2. Preranked GSEA was performed using the fgsea package (permutation‐based enrichment test, BH adjustment for multiple‐testing correction, v1.36.2) [68]. Gene sets were retrieved from the msigdbr package (v26.1.0) [69] and included the Hallmark and Gene Ontology Biological Process (GOBP) collections. Pathways were required to have a minimum of 15 and a maximum of 500 genes.

### 5.11 Culture Time Clustering Analysis

Temporal gene expression clustering was performed using the Mfuzz package (v2.70.0). Genes with low variance (standard deviation ≤ 0.5, calculated based on normalized data) were excluded. To determine the optimal number of clusters, k‐means clustering was applied with default parameters except for *nstart = 25* and with k ranging from 2 to 10 on median‐aggregated, normalized gene expression data summarized by week and culture time. The within‐cluster sum of squares and the average silhouette width (computed using the cluster package, v2.1.8.2) were used to determine the optimal number of clusters. Before being input into the mfuzz function, normalized gene expression values were standardized by gene to have a mean value of zero and a standard deviation of one (z‐scoring). The fuzzifier parameter (m) was estimated from the data. The five resulting gene clusters contained 450, 237, 341, 568, and 307 genes, respectively. Functional enrichment analysis on the genes in each resulting cluster was performed using gseapy (v1.1.11) [70] and the GOBP gene set collection [71].

### 5.12 Gene Set Scoring

Custom gene expression signatures for skin‐focused gene sets were quantified using a curated set of canonical marker genes (see Table S1). Marker genes for the SenSkin senescence gene signature were obtained from Wyles et al. [53]. Normalized expression values were z‐scored across samples to obtain relative expression levels. For each gene set, expression signatures were computed by averaging z‐scored expression values across all genes within the set. For gene set score comparisons between groups, the median gene set score of the reference group was subtracted from the median gene set score of the experimental group to obtain the gene set score difference. Statistical significance between groups was assessed using two‐sample t‐tests (two‐sided, unpaired Welch's t‐tests with BH correction for multiple testing).

### 5.13 Histology

At each harvest time point, sectioned biopsies were fixed in 10% formalin and shipped the same day to iHisto (Salem, MA, USA) for further processing. The fixed tissues were trimmed, processed and embedded in paraffin, followed by sectioning to 5 µm thickness. Sections were then stained with H&E and EVG for histological evaluation. IF staining was performed using primary antibodies against KRT5 (ab52635, Abcam, Cambridge, UK), KRT10 (ab76318, Abcam), and filaggrin (ab221155, Abcam). Whole‐slide images were captured using a PANNORAMIC 1000 digital scanner (3DHISTECH Kft., Budapest, Hungary) by the service provider (iHisto).

### 5.14 Image Analysis

H&E‐stained whole‐slide images were analyzed using QuPath (version 0.5.1) for histological assessment. IF images were visualized and analyzed for marker expression using SlideViewer (Version 2.9.0.229983, 3DHISTECH Ltd., Budapest, Hungary).

### 5.15 Biochemical Assays

The cytotoxicity levels in effluents were assessed by measuring LDH release using the CytoTox 96 Non‐Radioactive Cytotoxicity Assay kit (Promega, G1780, Madison, WI, USA), according to the manufacturer's instructions. Based on study design and requirements, effluents were analyzed for human IL‐8 (ELH‐IL8‐5), human IL‐6 (ELH‐IL6‐5), human MCP‐1 (ELH‐MCP1‐5), human MMP‐1 (ELH‐MMP1‐5) and human S100A8/A9 (ELH‐S100A8‐9‐5) using enzyme‐linked immunosorbent assay (ELISA) kits (RayBiotech, Inc., Norcross, GA, USA) in accordance with the manufacturer's protocols. The absorbance at 450 nm was detected using a Synergy H1 multimode microplate reader (Agilent Technologies, Santa Clara, CA, USA) and analyzed using the built‐in Gen5 software (Version 3.12).

### 5.16 Statistical Analysis

Non‐transcriptomic statistical analyses and graphs were produced using GraphPad Prism version 11.0.0 for Mac OS X, GraphPad Software, Boston, Massachusetts USA. For donor‐level statistical comparisons, donors were treated as biological units and technical replicates were averaged within each donor and experimental condition before statistical analysis. Depending on the experimental design, data were analyzed using one‐way repeated‐measures analysis of variance (ANOVA) or a mixed‐effects model, followed by Dunnett's multiple‐comparisons test for the specified treatment comparisons. Multi‐donor bar plot data are presented as mean ± standard error of the mean (SEM). For the non‐transcriptomic heatmap in Figure 3I, values represent mean protein concentrations aggregated across replicate measurements from multi‐donor experiments. The number of donors, technical replicates, statistical tests, and significance thresholds are specified in the corresponding figure legends. For transcriptomic analyses, differential gene expression was tested using the DESeq2 Wald test (two‐sided) with genes considered significant at a BH‐adjusted *p* < 0.05. GSEA was performed on DESeq2‐ranked genes using fgsea (permutation‐based enrichment test, FDR values reported). Gene set scoring comparisons (Figures 2E and 4G) were calculated using two‐sided, unpaired, Welch's t‐tests and corrected for multiple testing using BH. All RNA‐seq and downstream transcriptomic analyses were performed using R v4.5.2 (DGE, GSEA, batch correction, culturing time clustering analysis) and Python v3.14.3 (gene set scoring, gseapy‐based functional enrichment of Mfuzz clusters).

## Author Contributions

K.‐J.J. conceived, designed, and supervised the study, interpreted the data, generated the final figures, and wrote the manuscript. P.K.S. led experimental design and execution, with contributions from E.A., J.C., E.G., P.T., S.H., B.L.C., and J.M., and contributed to data visualization. E.S. performed RNA‐seq data analysis, generated associated figures, and contributed to writing the Results section. C.H. designed and manufactured the insert platform, optimized the tissue press workflow, and developed the data platform infrastructure. L.K. and S.Z. contributed to early model and application development. S.L. and J.H. supported experiments, scientific discussion, and analytical logistics. All authors contributed to the Methods section and reviewed the manuscript.

## Conflicts of Interest

All authors are employees of and/or hold equity in Outer Biosciences, Inc.

## Supporting information




**Supporting File**: advs78035‐sup‐0001‐SuppMat.docx.

## Data Availability

The data that support the findings of this study are available from the corresponding author upon reasonable request.
