# Supplementary material for: Long‐Term Human Skin Platform for Modeling Chronic Inflammation, Environmental Stress, and Therapeutic Intervention
Source: Adv Sci (Weinh). 2026 Sep 29:e78035. Online ahead of print. doi: 10.1002/advs.78035 (PMC13624414; doi:10.1002/advs.78035)
Supplement: Supplementary file 1 [file ADVS-9999-e78035-s001.docx]

Supporting Information

Long-Term Human Skin Platform for Modeling Chronic Inflammation, Environmental Stress, and Therapeutic Intervention

Purnendu Kumar Sharma^1†^, Evelien Schaafsma^1†^, Elysia Anderson^1^, Jocelyn Chiari^1^, Paige Thompson^1^, Erin Galt^1^, Sunghoon Lee^1^, Jason Holsapple^1^, Sai Hein^1^, Bailey L Coates^1^, Jade Michaud^1^, Sabrina Zuccaro^1^, Lia Kent^1^, Chris Hinojosa^1^, and Kyung-Jin Jang^1^*

^1^Outer Biosciences, Inc., Malden, MA, USA

†These authors contributed equally to this work.

*Corresponding Author, [kj@outerbio.com](mailto:kj@outerbio.com)

**Figure S1. Time-dependent comparison of tissue architecture in conventional Transwell and 3D-printed insert culture.**

Representative H&E images of donor-matched human skin biopsies cultured for 7 or 15 days using either a conventional Transwell-based configuration or the 3D-printed insert platform. Scale bar = 100 µm.

**Figure S2. Early senolytic-associated responses in aged human skin.**

(A) Quantification of IL-6 and MMP-1 levels following treatment with vehicle, dasatinib, or dasatinib plus quercetin (D+Q) at Week 1. No significant differences were observed between treatment groups. Data are presented as fold change relative to vehicle control. (B) GSEA of the same selected Hallmark pathways shown in Figure 4E at Week 1. None of the pathways reached the predefined significance threshold. Circle size indicates −log10(FDR), and color indicates NES. (C) Principal component analysis (PCA) of transcriptomic profiles colored by treatment and donor, indicating no clear separation between groups.

**Table S1. Curated compartment- and cell type-associated gene sets used for transcriptomic signature analysis.**

Gene sets representing epidermal, dermal/ECM, and immune-associated transcriptional programs used for longitudinal gene set scoring analyses.

**Table S2. Curated inflammaging gene set used for aging-associated transcriptional signature analysis.**

Inflammaging-associated genes used to quantify chronic inflammatory and aging-associated transcriptional programs in human skin tissues.
